# The contribution of domestic animals to the transmission of schistosomiasis japonica in the Lindu Subdistrict of the Central Sulawesi Province, Indonesia

**DOI:** 10.14202/vetworld.2019.1591-1598

**Published:** 2019-10-23

**Authors:** Novericko Ginger Budiono, Fadjar Satrija, Yusuf Ridwan, Ekowati Handharyani, Sri Murtini

**Affiliations:** 1 Parasitology and Medical Entomology Study Program, Graduate School, IPB University, Bogor, Indonesia; 2 Department of Animal Infectious Diseases and Veterinary Public Health, Faculty of Veterinary Medicine, IPB University, Bogor, Indonesia; 3 Department of Veterinary Clinic, Reproduction, and Pathology, Faculty of Veterinary Medicine, IPB University, Bogor, Indonesia

**Keywords:** coprology, mammalian animals, schistosomiasis, transmission, zoonosis

## Abstract

**Background and Aim:**

Schistosomiasis is endemic in Indonesia and is found in three remote areas in Central Sulawesi Province. Non-human mammals serve as reservoir hosts, meaning the disease is zoonotic. The previous schistosomiasis studies in animals from the Lindu Subdistrict did not determine which domestic animal species can serve as the primary source of transmission. No animals have been treated in Indonesia to control the disease; therefore, the parasite’s life cycle is not blocked entirely. This study aimed to determine the prevalence and identify the risk factors associated with, *Schistosoma japonicum* infection in animals, and identify animals’ relative contributions to *S. japonicum* transmission in the Lindu Subdistrict.

**Materials and Methods:**

A cross-sectional survey of *S. japonicum* infected animals was conducted in five villages of the Lindu Subdistrict. Fecal samples were collected from 134 selected animals (13 cattle, 26 buffaloes, 28 horses, 59 pigs, and 8 dogs). *S. japonicum* infection and infection intensity were determined using the Danish Bilharziasis Laboratory method. Environmental contamination with schistosome eggs was measured. The data were analyzed using a Chi-square test.

**Results:**

The overall prevalence of schistosomiasis was 32.9%, with the prevalence of infection in each species of animal at 61.5% in cattle, 42.3% in buffaloes, 25.0% in horses, 35.6% in pigs, and 12.5% in dogs. Free-range pigs were 8.667 times more likely to have *S. japonicum* infection than pigs kept in cages. Buffaloes, cattle, and horses were the primary sources of *S. japonicum* egg contamination, with relative transmission indices of 59.15%, 22.80%, and 10.61%, respectively.

**Conclusion:**

Bovines and horses are the main contributors to schistosomiasis transmission in the Lindu Subdistrict. In conjunction with other schistosomiasis control programs, the government should treat infected animals living within endemic areas where there are high infection rates of *S. japonicum*.

## Introduction

Schistosomiasis is a parasitic disease, which contributes to global public health and economic problems. Globally, about 250 million people have contracted the disease [1], and more than 800 million people are at risk of infection [2]. Helminth parasites from the genus of *Schistosoma* are the etiological agents. Six species cause infections in humans, namely, *Schistosoma mansoni*, *Schistosoma japonicum*, *Schistosoma haematobium*, *Schistosoma intercalatum*, *Schistosoma guineensis*, and *Schistosoma mekongi*, which are endemic in tropical and subtropical countries [3]. Schistosomiasis japonica, a term for infection by *S. japonicum*, can be found in Indonesia, the Philippines, and the People’s Republic of China. Infection by *S. japonicum* is zoonotic because, in addition to humans, domestic, and wild mammals can also act as reservoir hosts. The nature of such an unusual zoonotic disease complicates control measures [4]. The intermediate host of *S. japonicum* in Indonesia is the snail, from the species of *Oncomelania hupensis lindoensis* [4]. Infection by *S. japonicum* in humans causes anemia, growth disorders, disorders of chronic abdominal organs (portal vein enlargement and enlargement of the liver and spleen), fibrosis of the liver [5], and even death [6].

In Indonesia, 28 villages in two districts (Sigi District and Poso District) of the Central Sulawesi Province are endemic for schistosomiasis. The prevalence of human schistosomiasis in Indonesia has fluctuated in the past decade. The prevalence in the Napu Valley has decreased, but there is an upward trend overall [7]. Since 2005, a control program has led to a decrease in prevalence from 37% to 1% or less in Lindu and Napu, but in 2008-2011, the prevalence increased between 0.3 and 4.8% in Napu and 0.8 and 3.2% in Lindu [4]. The first reported infection with *S. japonicum* in Bada, in 2008, with a prevalence of 0.5%; a survey conducted in 2010 showed an increase in incidence to 5.9% [7]. Research on each mammalian species’ contribution to disease transmission by measuring the relative transmission index (RTI) is essential to developing a strategy for controlling schistosomiasis [8]. The previous studies in the Philippines and China have revealed that bovines are the primary source of schistosomiasis transmission [5,9], despite the differences in diagnostic tools used for fecal examination. A miracidia hatching test has been used routinely in China [10], and the Danish Bilharziasis Laboratory (DBL) technique has been used extensively in the Philippines [11] to detect *S. japonicum* infection among domestic animals. In the endemic areas of Indonesia, the significance of animals, especially domestic animals living close to the community, in schistosomiasis transmission is still unknown. As there is no vaccine for schistosomiasis, praziquantel is the primary treatment for infected humans and animal reservoir hosts [12].

The current study conducted with the aims to (1) determine the prevalence of schistosomiasis in animals, (2) identify risk factors for schistosomiasis in domestic animals, and (3) determine the relative roles of domestic animals in *S. japonicum* transmission in the Lindu Subdistrict, Province of Central Sulawesi, Indonesia.

## Materials and Methods

### Ethics approval and Informed consent

The authors obtained ethical clearance from the Animal Ethics Committee, Institute of Research and Community Development, IPB University No. 69-2017. Appropriate informed consent was obtained to collect animal stool samples, and animal sampling was performed using an approved protocol.

### Study design and study area

This cross-sectional study, conducted in August 2017, consisted of measuring the rate and the infection intensity of *S. japonicum*, associated risk factors, and the relative contribution of a number of animals in disease transmission. The animal owners were interviewed using a questionnaire to record potential risk factors. Geographically, the Lindu Subdistrict is located in 1°13’37”S until 1°30’15”S and 120°00’43”E until 120°17’17 “E. Topographically, the Lindu Subdistrict is upland and a valley, with a total area of 11962.5 Ha. The Lindu Subdistrict consists of five villages, namely, Puroo, Langko, Tomado, Anca, and Olu [13].

### Sample size, sample collection, and stool examination

The sample size was determined using the Thrusfield formula [14], with a confidence interval (CI) of 95%, an expected prevalence of 24.5%, and an error rate of 8%. Using this formula, the minimum sample size was determined to be 114. A total of 134 animal stool samples (from 13 cattle, 26 buffaloes, 28 horses, 59 pigs, and 8 dogs) were collected from five villages in the Lindu Subdistrict. The authors interviewed the animal owners to find out the characteristics of the owners and animals, such as age, sex, and state of care.

All animal owners were asked not to release the animals in the morning before the stool samples were collected. Stool samples were collected from the animals by veterinarians or trained veterinary paramedics. Each stool sample taken was labeled (location, code, animal species, sex, animal age, owner’s name, the animal husbandry management, and sampling date). Animal feces were taken as directly as possible, through rectal palpation or from individual cages, at a minimum of 20 g. Animal samples were obtained by limiting the animal to a pen or binding the animal until the samples were collected. If there was no individual animal cage, animals were tied, and then, the feces that were excreted the next morning were collected. The laboratory work was performed at the Helminthology Laboratory, Faculty of Veterinary Medicine, IPB University.

### DBL technique

The DBL technique was a combination technique involving filtration and sedimentation. Briefly, a total of 5 g of fecal matter were weighed, dissolved in 50 mL 0.9% NaCl, homogenized, and filtered using multilevel filters (filter sizes 400, 100, and 40 µm). The mixture of fecal materials retained in the 40 µm filter was put into a Baermann glass with 0.9% NaCl and left in a dark room for 10 min. The sediment was transferred to a test tube to be centrifuged and resuspended in 0.9% NaCl to a total volume of 2.25 mL. Then, 150 µL of the mixture was pipetted and added to 850 µL 0.9% NaCl to a total of 1 mL in the counting chamber. Eggs were counted 3 times to determine the number of eggs per gram of stool. If the number of eggs calculated between repeated measures was more than 10% different, or the stool size was smaller than 5 g, then the calculation involved dividing the total number of eggs estimated from three counting chambers by the weight of the actual sample and then multiplying by 5 [15].

### Measurement of environmental contamination parameters

#### Total daily egg output

The stool weight of each species was assumed based on the previous research [16,17]. In China and the Philippines, cattle and buffaloes can excrete 25-50 kg of feces per head every day. In this study, 25 kg was used as a conservative number for cattle and buffalo excretion. In Indonesia, the same data were accounted for cattle and buffalo. Horses were determined to excrete 10 kg/day [16]. Data regarding the prevalence and intensity of infection were used to calculate the level of contamination of feces with eggs based on the DBL method. Measurement of the total daily egg excretion (TDEE) for each animal species was conducted using the following formula:


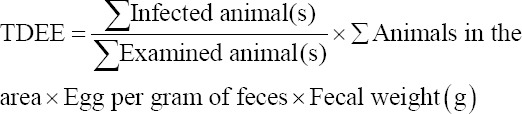


#### Animal contamination index (ACI)

The ACI for each species of animal was calculated using a formula that has been previously published [18]. ACI=Arithmetic mean of egg per gram of feces×∑ infected animal(s)×fecal weight (g).

#### RTI

The RTI was measured using the formula as follows [17]:


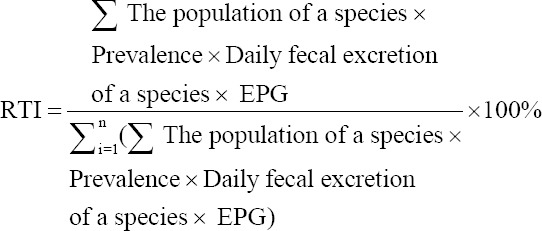


### Statistical analysis

Data were entered into Microsoft Excel 2010. A Chi-square test was used to determine the significance of risk factors (gender, age, and maintenance methods) with a significance value (α) of 0.05.

## Results

### Respondents

There were 38 animal owner respondents, most of whom (86.84%) were male. Nearly three-quarters of respondents were ≥40 years old. Only seven respondents were educated to at least high school or college level. Most respondents (89.47%) were farmers, and others were civil servants or traders.

### Prevalence of schistosomiasis in animals

The main species assessed were buffaloes (maintained for meat, working animals, and traditional events), cattle (meat and traditional events), pigs (meat and traditional events), and horses (working animals). The total number of animals consisted of cattle, buffaloes, horses, pigs, and dogs living in the Lindu Subdistrict. There were 134 animal fecal samples, including 59 pigs, 28 horses, 26 buffaloes, 13 cattle, and 8 dogs from five villages (Anca, Tomado, Puroo, Langko, and Olu villages). *S. japonicum* infection rate in cattle, buffalo, horses, dogs, and pigs is shown in Table-1. Overall, the schistosomiasis prevalence was 32.9% (95% CI 25.5-41.2%). *S. japonicum* prevalence was highest in cattle (61.5%; 95% CI 54.2-68.8%), while the lowest infection rate of *S. japonicum* was observed in dogs (12.5%; 95% CI 4.5-20.5%) (Table-1).

**Table 1:** Prevalence of *S. japonicum* infection in each species and village in the Lindu Subdistrict based on the Danish Bilharziasis Laboratory method.

Animal species	Number of positive animals/sampled animals						Prevalence in % (95% CI)

	Village					Total	

	Anca	Tomado	Langko	Puroo	Olu		
Cattle	7/11	-	-	-	1/2	8/13	61.5 (54.2-68.8)
Buffalo	0/2	0/4	11/20	-	-	11/26	43.3 (39.6-47.0)
Horse	-	-	7/28	-	-	7/28	25.0 (22.0-28.0)
Pig	0/18	0/1	10/15	4/16	7/9	21/59	35.6 (34.0-37.2)
Dog	-	1/5	0/3	-	-	1/8	12.5 (4.5-20.5)
Total	7/31	1/10	28/66	4/16	8/11	47/134	32.9 (25.5-41.2)

CI=Confidence interval, *S. japonicum*=*Schistosoma japonicum*

### Risk factors for schistosomiasis in animals

In pigs, the only significant risk factor of *S. japonicum* infection was animal husbandry management (p<0.000). The odds ratio of penned pigs versus free-roaming pigs was 8.667 (95% CI 2.510-29.929), which means that free-range pigs will probably be infected by *S. japonicum* 8.667 times more than pigs kept in cages (Table-2). Age and sex were not deemed as risk factors for schistosomiasis in pigs (both p>0.05). All of the cattle sampled were adults and were kept in cages at night. However, from morning until the evening, they were allowed to graze in the grazing area. The prevalence of male cattle was slightly higher (66.67%) than female (60.00%), but sex was not determined as a risk factor of schistosomiasis in cattle as this difference was not statistically significant (p>0.05). Similar to cattle, all buffaloes sampled were kept in cages at night and released to the grazing area during the day. Buffalo calves had a higher prevalence (35.71%) than adult buffalo (25.00%), but this difference was not statistically significant (p>0.05). The prevalence of schistosomiasis in male buffalo (41.67%) was higher than the prevalence in female buffalo (22.73%), but this was also not significantly different (p>0.05). All of the horses sampled in the study were adults. The horses were free-range. No male horse was identified as infected by *S. japonicum*, while 28.00% of female horses were positive. However, statistically, sex was not a significant risk factor for schistosomiasis in horses (p>0.05). No risk factors were identified as significant in dogs (p>0.05).

**Table 2:** The prevalence, intensity of infection (arithmetic and the geometric mean of eggs per gram of feces), and animal contamination index of *S. japonicum* in animals in the Lindu Subdistrict.

Species	Number of samples	Number of positive samples	Prevalence in percentage (95% CI)	The arithmetic mean of egg per gram of feces	The geometric mean of egg per gram of feces	Animal contamination index
Cattle	13	8	61.5 (54.2-68.8)	8.6	5.7	1,720,000
Buffalo	26	11	43.3 (39.6-47.0)	7.2	3.6	1,980,000
Horse	28	7	25.0 (22.0-28.0)	25.2	7.0	1,764,000
Pig	59	21	35.6 (34.0-37.2)	16.4	6.9	172,200
Dog	8	1	12.5 (4.50-20.50)	2.0	2.0	300

CI=Confidence interval, *S. japonicum*=*Schistosoma japonicum*

**Figure-1:**
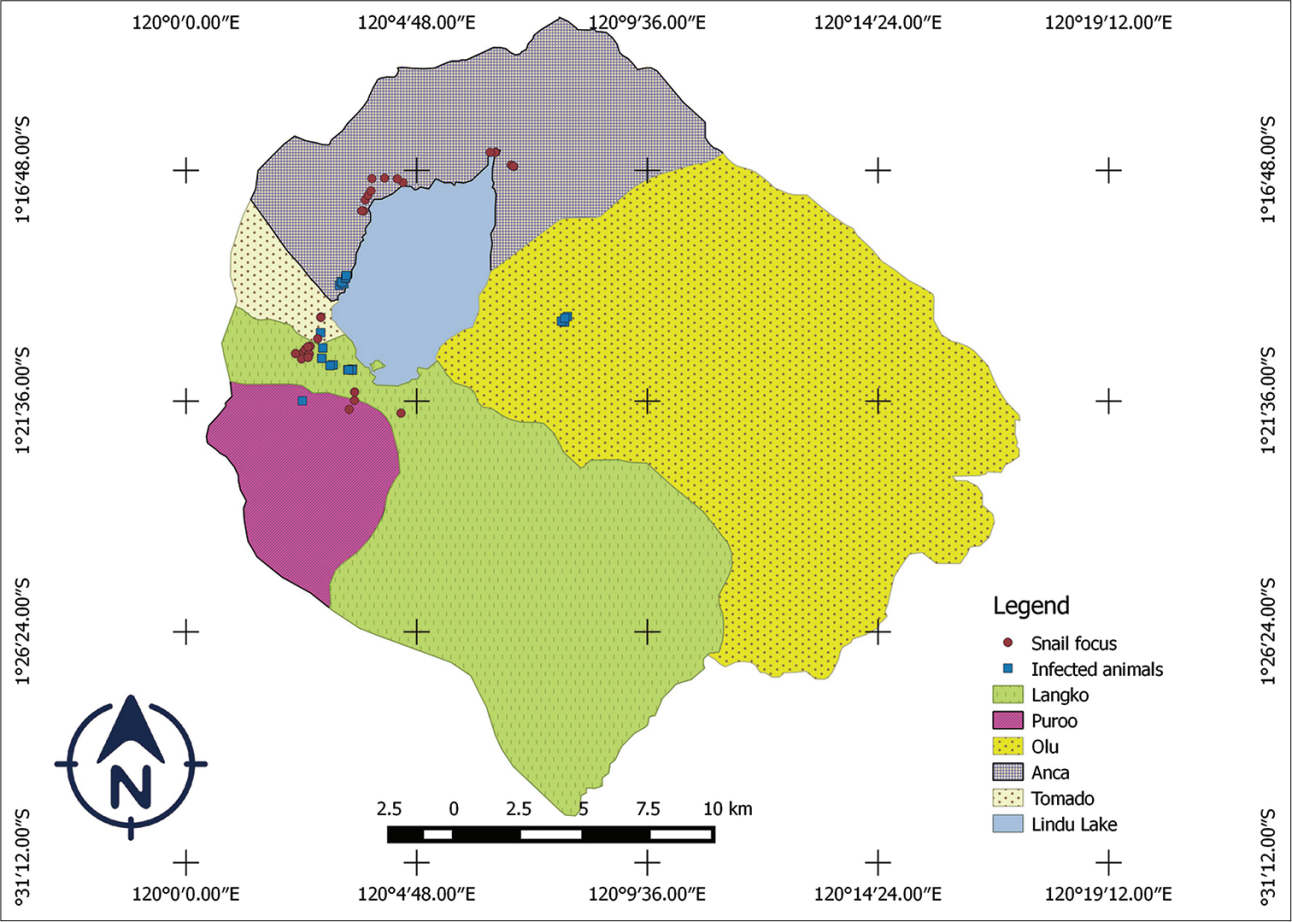
Map of schistosomiasis japonica spread among animals in the Lindu Subdistrict. [Source: Map was illustrated by Novericko Ginger Budiono]

As shown in Table-2, the infection intensity was highest in horses (the arithmetic and the geometric mean of eggs per gram feces were 25.2 and 7.0, respectively). Dogs had the lowest arithmetic and geometric mean values for eggs per gram of feces of 2.0 and 2.0, respectively. The results of the mapping regarding the distribution of animals infected with *S. japonicum* in the Lindu Subdistrict are shown in Figure-1. Figure-1 shows the distribution of animals suffering from *S. japonicum* infection and their overlapping host range with the *O. hupensis lindoensis* snail foci.

### Contamination index for host species

The mean daily mass of excreted feces per host each species has been published previously [17]. The ACI (the total number of daily eggs discharged) was, 1,720,000, 1,980,000, 1,764,000, 172,200, and 300, for cattle, buffaloes, horses, pigs, and dogs, respectively (Table-2).

The population of cattle, buffalo, horses, dogs, and pigs was obtained from the Sigi district Animal Husbandry and Animal Health Service (2017) [19]. In this study, the survey coverage (i.e. the number of animals sampled divided by the total population) of cattle, buffalo, horses, dogs, and pigs were 7.6%, 4.5%, 16.7%, 4.3%, and 7.0%, respectively. There were 94,640,883 eggs released by all infected animals each day. Table-3 shows the RTI, which indicates each species role in the transmission of *S. japonicum* infection. The data on eggs per gram of feces shown in Table-3 are based on the DBL technique. The relative contribution of buffaloes in schistosomiasis transmission in the Lindu Subdistrict was the greatest (62.3%) followed by cattle (24.03%) and horses (11.18%). The contributions of pigs and dogs in *S. japonicum* transmission were not significant, with an RTI of only 2.39% and 0.05%, respectively (Table-3). The calculations indicated that 94,640,883 *S. japonicum* eggs were released every day by all of the animals in the Subdistrict of Lindu (Table-3).

**Table 3:** Total daily egg excretion and relative transmission index of *S. japonicum* in the Subdistrict of Lindu.

Animal species	Number of animal population	Number of animals examined	Number of positive animals	Prevalence (%)	The arithmetic mean of egg per gram of feces	Weight of feces (g)	Total daily egg excretion	Relative transmission index %)
Cattle	172	13	8	61.5	8.6	25,000	22,742,700	24.03
Buffalo	757	26	11	43.3	7.2	25,000	59,000,580	62.34
Horse	168	28	7	25	25.2	10,000	10,584,000	11.18
Pig	842	59	21	35.6	16.4	500	2,263,128	2.39
Dog	1346	8	1	12.5	2	150	50,475	0.05
Total	3285	134	47	32.9			94,640,883	

*S. japonicum*=*Schistosoma japonicum*

## Discussion

Since 1937, the Lindu Subdistrict has been known as an endemic area for schistosomiasis japonica. Brug and Tesch were the first to report the presence of *S. japonicum* eggs in autopsied human bodies in Palu Hospital [4]. There is still disease transmission in this area. In Indonesia, the surveillance of schistosomiasis in endemic areas, including the Lindu Subdistrict, is performed twice a year to measure the prevalence of schistosomiasis in humans, snails (*O. hupensis lindoensis*), and rodents (as reservoir hosts). Unfortunately, schistosomiasis surveillance in mammals (other than humans and rodents) is not continuous. There is particular concern regarding schistosomiasis because it is zoonotic, meaning that non-mammalian hosts can also act as reservoir hosts when they are infected with *S. japonicum*. For instance, reports of infections in mammals other than rodents in the Lindu Subdistrict were recently reported by Gunawan *et al*. [20]. Budiono *et al*. [21] also indicated the presence of trematode infections, including *S. japonicum*, in cattle and buffaloes in Lindu and other endemic locations. According to the author’s knowledge, no one has reported the RTI of each mammalian species (humans and other mammals) in the Lindu Subdistrict.

The primary animal commodities in the Lindu Subdistrict are buffaloes, pigs, and beef cattle. Although there are few reports of schistosomiasis infection in animals, there have been no specific interventions to provide treatment (in the form of praziquantel) for infected animals. Therefore, there is still a knowledge gap that needs to be filled with research aimed at investigating the prevalence of schistosomiasis in animals and their relative contribution to transmitting the disease to humans. This is a pilot study that seeks to answer the hypothesis that domestic animals help spread schistosomiasis in humans in the Lindu Subdistrict. The DBL technique was used to detect the presence of *S. japonicum* eggs in domestic animal feces.

This study reported that the overall *S. japonicum* infection in five domestic animal species in the Lindu Subdistrict was 32.9% (95% CI 25.5-41.2%). The prevalence of schistosomiasis in this study is slightly higher than a previous report by Gunawan *et al*. [20], amounting to 24.66% from a total of 219 animal samples infected by *S. japonicum*. This difference in prevalence may be due to differences in the examination methods used. Gunawan *et al*. [20] conducted the *S. japonicum* infection survey in 2013 with a formalin-ether sedimentation testing technique, a different sample size, and a different survey time. This result was also in contrast with a previous study by Izhar *et al*. [22], which reported that there was no *S. japonicum* infection among 202 animals (water buffaloes, horses, dogs, pigs, and cows) examined in the Lindu Subdistrict.

There has been a fluctuation in the prevalence of schistosomiasis in humans in the Lindu Highlands in recent years (between 2012 and 2017), fluctuating from 1.22%, 0.72%, 0.90%, 1.24%, 0.92%, to 0.85%, from year to year, respectively [23]. Repeated infections can contribute to this fluctuation. In each village, the prevalence of human schistosomiasis in 2017 was 0.69% in Anca, 0.37% in Tomado, 2.14% in Puroo, 0.73% in Langko, and 0.36% in Olu [23].

In 2016, *S. japonicum* infection in the *O. hupensis lindoensis* snail, sampled from the Lindu Subdistrict, varied from 1.2 to 14.5% [23]. In recent studies [24,25], it was reported that in the Anca Village of the Subdistrict of Lindu, a total of 12 O. *hupen­sis lindoensis* snail foci were present with a total area of 19,784 km^2^. In the Langko village, there was one focus of *O. hupensis lindoensis* snail that was found with a cumulative area of 6886 km^2^. In the village of Puroo, there were three *O. hupensis lindoensis* snail foci with a total area of 487,546 km^2^, while in the Olu village, no *O. hupensis lindoensis* snail focus found. Based on schistosomiasis distribution maps reported by Sudomo and Carney [8], the area which is now known as Olu is an endemic area for schistosomiasis. This is shown in Figure-1, where the locations that animals are likely to be infected by *S. japonicum* are around the focus of the *O. hupensis lindoensis* snail. Further study is needed to identify the presence of current snail foci in the Olu village.

This study uses the assumptions of daily body weight released by each animal species at the study site based on the previous research [16,17] regarding the total stool weight released per day by each species. It would be better if measurements were taken directly from each animal species at the study site. Researchers may find it difficult to measure some free-range animals.

The DBL technique was used to determine *S. japonicum* infection in a wide range of animals, namely, cattle, horses, buffaloes, dogs, cats, and rats, with high specificity (>92%) and high sensitivity (80-96%) [15,26]. The advantages of the DBL technique are that it is (1) easy to apply; (2) a quantitative diagnostic test; and (3) non-toxic; it (4) can distinguish living and dead eggs; (5) can be reread for quality control; and (6) can be used to diagnose infections by other trematodes such as *Fasciola gigantica*, *Paramphistome*, *Dicrocoelium dendriticum*, and *Plagiorchis javensis* [21,26-28].

In the present study, the DBL technique was used as described by Carabin *et al*. [26] for detecting *S. japonicum* infection in a range of animal species. Further research is necessary to identify each animal species’ actual contribution to schistosomiasis transmission, such as miracidia hatchability and the actual defecation of animals in snail foci. A previous study has also reported that each different animal species tended to excrete a different hatching rate of *S. japonicum*. It would be better if the amount of *S. japonicum* miracidia hatched per gram of stool could be determined rather than the number of *S. japonicum* eggs per gram of stool [17]. The infection rate of *S. japonicum* in all domestic mammal species in the Subdistrict of Lindu is still relatively high (>5%).

Infected animals are a vital source of infection and play a role in spreading the disease. In each particular endemic area, each mammal species is considered to play a respective role in *S. japonicum* transmission. Domestic animals residing in endemic areas are susceptible to *S. japonicum* infection, but their relative contribution to the spread of *S. japonicum* eggs may be different. The leading causes of the spread of eggs are environmental factors and management practice. Studies in China revealed that, in the highlands, nocturnal rodents are the predominant hosts. The intermediate hosts shed the cercariae in the evening, while in the lowlands, bovines are the main shedders of *S. japonicum* eggs in the early morning when they come into contact with water [29]. In general, water buffaloes expelled more feces compared with cattle and other species. Therefore, water buffaloes are considered the most significant contributors to disease transmission [17]. The high prevalence of schistosomiasis in large livestock is affected by the animals’ continuous exposure to *S. japonicum* cercariae infections in the snail foci locations as a result of free-grazing patterns and the lack of praziquantel treatment.

This study succeeded in showing that large ruminants, namely, buffaloes and cattle, also played an essential role in disseminating *S. japonicum* eggs to the environment. This was evidenced by the deposition of 62.3% and 24.3% *S. japonicum* eggs by buffaloes and cows, respectively, in the Lindu Highland. Cattle and buffaloes are bred as animal protein sources and as working animals for agricultural purposes. The animal owners let the livestock, especially ruminants, to graze in disease transmission areas. This study’s results support the results of the previous research in China that cattle, buffaloes, and goats are the animals most involved in schistosomiasis japonica transmission [17,30-36]. Bovines have been considered one of the main contributors of *S. japonicum* transmission in China because they live in large populations, have long life spans, are free-roaming, and can excrete a high number of eggs into the environment [9]. This study also noted the importance of horses as potential *S. japonicum* reservoir hosts as schistosomiasis prevalence in the horse was found to be relatively high (25.0%) (95% CI 22.0-28.0%). The results show that these animals have an important role in the spread of *S. japonicum* infection to humans in Indonesia. Infection of bovines and horses by *S. japonicum* can occur while they graze or drink around ditches or water bodies contaminated by *S. japonicum* cercariae. The use of untreated animal excrement as agricultural fertilizer can also be an indirect source of contamination of *S. japonicum* eggs in the field. Biogas treatment, which creates an anaerobic atmosphere, of animal stool samples can prevent *S. japonicum* egg contamination due to a lack of oxygen.

Another potential host of *S. japonicum* is the pig. Our results show that 21 of 59 pigs (35.6%) were infected with *S. japonicum*. Pigs in the Lindu Subdistrict are mostly retained in pens, which reduces the risk of *S. japonicum* infection. This was identified in this study where pigs that were reared in a free-range environment were 8.667 times more susceptible to *S. japonicum* compared with pigs kept in cages. This, therefore, suggests that the rearing of pigs in a free-range environment is a significant risk factor in *S. japonicum* infection. This is in agreement with another study that concluded that penned animals could reduce transmission potential [17].

Our study reported that one of eight dogs tested were infected with *S. japonicum*. Infection of dogs with *S. japonicum* has rarely been studied as it is difficult to sample the stools from this species. The present study reported that dogs are not essential contributors to schistosomiasis transmission, justified by low rates of *S. japonicum* infection compared to other species, but there was still >5% transmission rate in dogs, which is still significant. The low rates of infection in dogs may be caused by limited contact with contaminated water. Another study by Wang *et al*. [17] also reported a low prevalence of schistosomiasis in dogs. As dogs in the Lindu Subdistrict are free-range, they can probably be infected by *S. japonicum* while drinking from or defecating in, small water bodies or ditches.

In the Philippines, there is a paradigm shift that buffaloes were thought to previously be considered insignificant in the spread of schistosomiasis japonica, but based on recent findings, it has now been agreed that they play an essential role in transmission [18]. In the Western Samar Province, dogs and rats were known to have a role in the transmission of *S. japonicum* into humans. Rats were also used as sentinel animals to determine the spread of schistosomiasis japonica in an endemic area [15].

The high prevalence of schistosomiasis japonica in animals in Lindu in this study shows the need for an integrated intervention program in animals. Control measures in China proved that the mass administration of praziquantel to animals could reduce *S. japonicum* infection in both humans and animals [9], but it cannot inhibit recurring infection. Developing a schistosomiasis control program requires a database, wherein relevant information regarding disease transmission dynamics is contained. Therefore, an approach is needed to be able to determine the level of infection between the intermediate host and the definitive host [37]. This study is the first step in understanding the level of schistosomiasis japonica in domestic animals as a definitive host separate from humans. This study can complement a database, considering that, schistosomiasis surveillance in domestic animals is still limited and not continuous. Besides, we need to integrate intervention in the form of praziquantel chemotherapy in animals, combined with other schistosomiasis control programs, and to reduce infection rates and control schistosomiasis in Lindu. To date, in Indonesia, there has never been an intervention trial with the administration of praziquantel drugs to provide treatment to animals. This research will be beneficial as a basis for developing schistosomiasis control programs in the future.

## Conclusion

The prevalence of schistosomiasis japonica in animals in the Lindu Subdistrict is high. In pigs, free-range rearing is a significant risk factor for infection with *S. japonicum*. This study concluded that cattle, buffalo, and horses were the primary source of *S. japonicum* infection in the Subdistrict of Lindu. In addition to other control measures, in endemic areas, the treatment of animals, which act as *S. japonicum* reservoir hosts, with praziquantel, is needed, especially in the Subdistrict of Lindu, to achieve the goal of schistosomiasis eradication by 2025. Further study is also needed to understand the number of hatched *S. japonicum* eggs per gram of feces.

## Authors’ Contributions

FS designed the study. NGB conducted the experiment, analyzed the data, and wrote the first draft of the manuscript. FS, YR, EH, and SM contributed to the drafting and revision of the manuscript. All authors read and approved the final manuscript.
